# Uveitis and Gender: The Course of Uveitis in Pregnancy

**DOI:** 10.1155/2014/401915

**Published:** 2014-01-09

**Authors:** Nathalie P. Y. Chiam, Lyndell L. P. Lim

**Affiliations:** Centre for Eye Research Australia, University of Melbourne, Royal Victorian Eye and Ear Hospital, 32 Gisborne Street, East Melbourne, VIC 3002, Australia

## Abstract

The hormonal and immunological changes in pregnancy have a key role in maintaining maternal tolerance of the semiallogeneic foetus. These pregnancy-associated changes may also influence the course of maternal autoimmune diseases. Noninfectious uveitis tends to improve during pregnancy. Specifically, uveitis activity tends to ameliorate from the second trimester onwards, with the third trimester being associated with the lowest disease activity. The mechanism behind this phenomenon is likely to be multifactorial and complex. Possible mechanisms include Th1/Th2 immunomodulation, regulatory T-cell phenotype plasticity, and immunosuppressive cytokines. This clearly has management implications for patients with chronic sight threatening disease requiring systemic treatment, as most medications are not recommended during pregnancy due to lack of safety data or proven teratogenicity. Given that uveitis activity is expected to decrease in pregnancy, systemic immunosuppressants could be tapered during pregnancy in these patients, with flare-ups being managed with local corticosteroids till delivery. In the postpartum period, as uveitis activity is expected to rebound, patients should be reviewed closely and systemic medications recommenced, depending on uveitis activity and the patient's breastfeeding status. This review highlights the current understanding of the course of uveitis in pregnancy and its management to help guide clinicians in managing their uveitis patients during this special time in life.

## 1. Introduction

Pregnancy is associated with various hormonal and immunological changes that facilitate the survival of the semiallogeneic foetus. These physiological changes influence the course of various maternal autoimmune diseases [1, 2]. The effect of pregnancy on noninfectious uveitis has not been as extensively studied; however, to date it has been well described by a few authors. It is essential to understand the course of uveitis in pregnancy as uveitis has a peak incidence in young adults and it is not uncommon for female patients with known uveitis to become pregnant. This review will examine the literature on the course of uveitis in pregnancy and its management. This summary would hopefully help guide clinicians in the management of uveitis during pregnancy and the postpartum period.

## 2. Theories on How Pregnancy Influences Uveitis

During pregnancy, the tolerance of the semiallogeneic foetus is made possible by the various hormonal and immunological changes in pregnancy. These physiological changes also have a role in influencing the course of maternal autoimmune diseases [1, 2].

The increased levels of oestrogen and progesterone during pregnancy result in the suppression of Th1 associated immunity but the upregulation of Th2 associated immune responses [3–5]. As such, pregnancy often ameliorates Th1 associated autoimmune diseases, like rheumatoid arthritis, but exacerbates Th2 associated autoimmune conditions, like systemic lupus erythematosus [2–9]. The association between uveitis amelioration and Th1 suppression/Th2 upregulation has been demonstrated by serum studies in Chan et al.'s [10] prospective case study on four pregnant uveitis patients. Agarwal et al. [11] have also reported similar findings for experimental autoimmune uveitis (EAU) in mice. When EAU susceptible mice (C57BL/6) were immunised with interphotoreceptor retinoid binding protein, the incidence and severity of EAU were lower in the pregnant mice, as compared to nonpregnant controls. The pregnant mice were also found to have reduced levels of interferon gamma, IL 12 P40 but unchanged levels of TNF alpha, IL4, IL5, and IL10, which suggested a Th2 bias in their immune system [11]. This Th2 bias in pregnancy probably augments the Th1 predominant response in noninfectious uveitis, resulting in disease amelioration [12]. Although still uncertain, the recently discovered subset of T helper cells, Th17, may also play a role in altered autoimmune activity in pregnancy [13–17]. Th17 cells are proinflammatory and associated with the pathogenesis of autoimmune diseases like systemic lupus erythematosus [18],Vogt-Koyanagi-Harada (VKH) disease [19], irritable bowel disease [20], rheumatoid arthritis [21], and multiple sclerosis [22]. During pregnancy, Th17 cells are elevated in preeclampsia [9, 23]. The hormonal and associated cytokine changes in pregnancy influence autoimmune disease activity and may inspire future therapeutic options. Interestingly, studies have shown that oral oestradiol may decrease disease activity in multiple sclerosis [24, 25]; however, its implications in uveitis management are uncertain.

Several other pregnancy-associated changes may influence the course of maternal autoimmune conditions. For instance, regulatory T cells demonstrate phenotype plasticity and are able to switch between a tolerant or aggressive phenotype in response to circulating foetal cells or infectious agents accordingly [17, 26]. The elevated levels of immunosuppressive cytokines and hormones, such as melanocyte-stimulating hormone [27, 28], early pregnancy factor [29], and alpha-fetoprotein [30, 31] have also been implicated in the improvement of various autoimmune conditions during pregnancy. The mechanism for altered activity of autoimmune uveitis in pregnancy is likely to be multifactorial.

The available literature seems to suggest that uveitis activity begins to improve in mid pregnancy and reaches its lowest level in the third trimester (see below). This may be due to the various pregnancy-associated changes, such as the Th1/Th2 immune shift, becoming increasingly pronounced with the progress of pregnancy [6, 32]. These findings are in keeping with the accepted theory that most forms of non-infectious uveitis are Th1 mediated diseases [12]. After delivery, the rate of flare-up seems to return to prepregnancy levels. This may be explained by the reversal of various pregnancy-associated changes within one to two months of delivery [33].

## 3. The Effect of Pregnancy on the Course of Uveitis

There have only been a few studies that investigated pregnancy's effect on noninfectious uveitis. Previous publications on uveitis in pregnancy include a few case reports [10, 34–36], a retrospective case series by Rabiah and Vitale [37] in 2003, and a retrospective cohort study by Kump et al. [38] in 2006. The authors of this review have also recently conducted a retrospective case series on uveitis in pregnancy [39]. As uveitis is an uncommon condition [40], studies on uveitis in pregnancy are constrained by the limited number of eligible patients and are largely restricted to retrospective studies. The general consensus is that uveitis activity improves in pregnancy, with significantly decreased disease activity from the mid pregnancy onwards. However, in the postpartum period, uveitis activity tends to relapse.

The findings from previous case reports and small case series (*n* ≤ 4) [10, 34–36] have limited generalizability due to the small numbers of patients studied. Even so, they reported that uveitis improves in pregnancy, especially in the mid and late trimesters while postpartum period was associated with activity relapse, which was reflected by other larger studies.

The retrospective case series by Rabiah and Vitale [37] was based in Saudi Arabia. It included 76 pregnancies among 50 women. Their subjects had VKH associated uveitis (46%), Behcet's disease associated uveitis (20%), and idiopathic uveitis (34%), which reflected the regional epidemiology in Saudi Arabia. The study investigated the probability of at least one flare-up in the periods three months before pregnancy, during pregnancy, and up to six months postpartum. They reported that the probability of uveitis flaring-up was lower during pregnancy as compared to three months pre-pregnancy and six months postpartum. It should be noted that the duration of followup in prepregnancy, pregnancy, and postpartum was unequal. As such, a larger number of patients may experience a flare-up when the duration of followup was longer; thus their findings should be interpreted with this in mind.

The retrospective cohort study by Kump et al. [38] was based in the United States of America. It involved 32 pregnant self-controls and 32 nonpregnant female controls who were matched for age, ethnicity, and anatomical location of uveitis. Most subjects had idiopathic uveitis (72%). They reported that the annual rate of flare-up was significantly lower during pregnancy (1.0 per year) as compared to nonpregnant periods (2.4 per year) and non-pregnant controls (3.1 per year), *P* < 0.001. During pregnancy, rates of flare-up decreased significantly in the second and third trimester (2.3, 0.5, 0.4 per year for the first, second, and third trimesters, resp.).

Chiam et al.'s retrospective study was based in Australia and included 47 subjects [39]. Uveitis activity one year prepregnancy, during pregnancy and one year postpartum was evaluated. The reported flare-up rates were 1.188, 0.540, 0.972 per person year in prepregnancy, gestation, and postpartum, respectively. (*P* < 0.001 for comparison between pre-pregnancy and pregnancy; *P* = 0.009 for comparison between pregnancy and postpartum). The rate of flare-up was 1.188, 0.264, 0.096 per person year for the first, second, and third trimesters, respectively. Rates in the second trimester were significantly lower than rates in the first trimester, *P* = 0.002; meanwhile rates in the third trimester did not differ significantly from the second trimester, *P* = 0.338. After delivery, rates of flare-up rebounded, as flare-up rates six months postpartum were not significantly different from pre-pregnancy rates (*P* = 0.306).

Interestingly, the severity of uveitis flare-ups does not seem to be influenced by the course of pregnancy. Chiam et al. reported that when uveitis severity was evaluated based on anterior chamber cell count, the severity of flare-ups was not significantly different between pregnancy and nonpregnant periods [39]. In Rabiah and Vitale's study [37], surrogate markers of disease severity including flare-up duration and type of therapy prescribed were also not significantly different in pregnancy and nonpregnant periods.

Other factors have also been studied with regard to their possible influence on uveitis activity during this period. These include the effect of breastfeeding, the possible relationship between multiple pregnancies in the same individual and various host factors such as type of uveitis.

Lactation has been suggested to aggravate some autoimmune diseases. After delivery, elevated prolactin levels from pregnancy will decline unless breastfeeding occurs. As prolactin is a proinflammatory hormone that promotes Th1-immune responses [2], Th1-dominant immunopathologies like rheumatoid arthritis have been shown to be aggravated by lactation [8, 41–45]. Although breastfeeding has not been found to have a significant influence on the likelihood of uveitis flare-up in the postpartum period, this is likely to be due to the small numbers of subjects available for analysis [37–39]. Similarly, although uveitis activity in pregnancy does not seem to be correlated between different pregnancies within multiparous individuals [37, 39], the small numbers of subjects available for analysis in these studies were again limited.

In general, the course of uveitis varies across uveitis aetiologies. However, it is interesting to note that in our study, host variables such as uveitis aetiology, anatomical location of uveitis, course of uveitis activity, medication used, and sex of child were not found to be associated with flare-up rates in pre-pregnancy, gestation, or postpartum period. In particular, it is interesting to note that uveitis activity seems to improve during pregnancy across most uveitis aetiologies. This is supported by other studies that analysed the effect of pregnancy according to uveitis diagnosis, where uveitis activity was found to improve from the second trimester onwards across the various uveitis aetiologies [37, 39]. Uveitis aetiologies analysed in these studies included HLA-B27 associated uveitis, VKH disease, Behcet's disease, and idiopathic uveitis.

Articles focusing on systemic autoimmune diseases in pregnancy have also suggested that the associated uveitis tends to improve for most of these conditions [3, 4, 7, 41, 42, 46–52]; however, the opposite applies to systemic lupus erythematosus, where ocular inflammation has been reported to increase in pregnancy [7, 51]. Meanwhile reports have been contradictory for VKH associated uveitis [53–58]. Rabiah and Vitale's retrospective study reported that their VKH subjects (*n* = 33) mostly experienced an early pregnancy flare-up, with approximately half experiencing a postpartum flare-up [37]. However, this has not been a consistent pattern amongst prior studies. Two case reports have described VKH patients experiencing flare-ups in mid and late pregnancy [55, 58]. Meanwhile, other case reports have described VKH activity in early pregnancy, with cases of VKH being first diagnosed between 10 and 16 weeks [56, 57]. There have also been case reports on VKH generally improving during pregnancy [53, 54]. It is therefore difficult to ascertain the course of VKH on pregnancy as the existing literature is restricted to case reports which describe inconsistent experiences.

The use of anti-inflammatory medications has not been found to be associated with rates of flare-up during pregnancy [37, 39]. However, this may be due to selection bias, as patients who did not receive treatment probably had relatively inactive uveitis, whereas those on medication likely had more aggressive disease that required treatment. On the other hand, the lack of association could also be due to the relatively small sample sizes (type II error) in these studies.

## 4. The Management of Uveitis in Pregnancy

The management of non-infectious uveitis in pregnancy attracts special interest as non-infectious uveitis is often managed with immunosuppressive agents that may affect fertility and the viability of pregnancies. The management of uveitis in pregnant women is therefore an area of uncertainty for clinicians due to the limited information available.

Wakefield et al. [59] recently published a review on the treatment of severe inflammatory eye disease in pregnancy and young patients of reproductive age. They advised that both male and female patients should be informed about the risks of infertility, miscarriage, and foetal abnormalities. Measures to address these adverse effects of immunosuppressants include sperm banking for male patients, oocyte cryopreservation for female patients, the use of double contraception (barrier and hormonal), and enforcing a drug washout period before conception is attempted. Female patients who become pregnant should be encouraged to inform their doctors as soon as possible so that their treatment may be modified if required for the safety of the pregnancy [59]. In general, principles in the management of uveitis in pregnancy include collaboration between the obstetrician, ophthalmologist, and the patient to evaluate the risks and benefit for the mother and child [59, 60].

Although many immunosuppressive agents are not recommended during pregnancy due to the lack of safety data rather than due to proven teratogenicity, some have proven adverse effects on the fetus and must be avoided. Specifically, methotrexate is contraindicated during pregnancy and lactation, as it results in both miscarriage and fetal anomalies. Similarly, cyclophosphamide and mycophenolate mofetil (MMF) should also be avoided in pregnancy. MMF has been associated with a high rate of fetal anomalies and miscarriages and has therefore resulted in the development of a risk evaluation and mitigation strategy (REMS) for this drug as mandated by the Food and Drug Administration [61]. Cyclophosphamide use poses fetal malformation risks and developmental delay and is absolutely contraindicated in early pregnancy [62, 63]. Although azathioprine and cyclosporine can be used with caution during pregnancy [63], there is currently insufficient data regarding the use of tumour necrosis factor blockers, anakinra and rituximab in pregnancy and lactation [59, 62, 63].  Table 1 summarises the current recommendations regarding the use of various immunosuppressive drugs in pregnancy, as advised in previous reviews [59, 62–65].

Wakefield et al.'s review proposed a stepwise therapeutic regimen for the management of uveitis in pregnancy according to disease severity. In mild uveitis, treatment could consist of topical or local steroid injections, followed by oral prednisolone (<50 mg/day), azathioprine (2 mg/kg/day), or cyclosporine (2.5–5 mg/kg/day). Higher doses of prednisolone (1 mg/kg/day) were recommended for more severe uveitis, with the addition of azathioprine and/or cyclosporine if needed. In the event where triple therapy with steroids, azathioprine, and cyclosporine was insufficient for the control of inflammation, the addition of intravenous immunoglobulin therapy or biological agents could then be considered [59]. In addition, for those patients taking chronic corticosteroids during pregnancy, Wakefield et al. recommended that the dose should be increased prior to delivery (24, 12, and 1 hour prior to delivery) to counteract the stress of childbirth.

However, as we and others have found that uveitis is generally less active during pregnancy than during the pre-pregnancy and postpartum periods, given the questionable safety of several systemic agents used in the treatment of non-infectious uveitis, an alternative approach would be to taper and/or cease systemic treatments during pregnancy in favour of locally delivered treatment.

The use of locally delivered treatment (such as periocular sustained release corticosteroid injections or intravitreal steroids) in non-infectious uveitis is not new and its use has been extensively described in a large range of non-infectious ocular inflammatory conditions. Periocular corticosteroids of triamcinolone and methylprednisolone have been effective in managing vitritis, posterior segment inflammation, and moderate macular oedema [66–73]. They confer the advantages of achieving higher drug levels in the posterior segment of the eye as compared to systemic steroids and lower risks of systemic side effects [74]. However, potential complications include ptosis, orbital fat protrusion, and other steroid induced ocular complications such as cataracts and raised intraocular pressure (IOP) [66, 73, 75, 76].

Intravitreal triamcinolone acetate (IVTA) is commonly used to treat vitritis and associated cystoid macular oedema [77–82]. Specifically, IVTA has been effectively used to treat uveitis associated with Behcet's disease [83–85], VKH syndrome [86], serpinginous choroiditis [87], and sympathetic ophthalmia [88–91]. These studies have shown that IVTA may be used alone or as an adjuvant to reduce the dose of systemic immunosuppression required. As compared to other forms of steroids, IVTA has been shown to be more effective than orbital floor and sub-Tenon triamcinolone [92, 93] and comparably as effective as oral steroids in managing posterior uveitis. However, side effects associated with IVTA include relatively high risks of steroid induced cataracts (15–30%) and IOP rise (25–45%), particularly in younger patients [81, 94]. This should be kept in mind when considering regionally delivered corticosteroids in uveitis patients during pregnancy. Other less common side effects include postinjection infectious endophthalmitis, pseudoendophthalmitis, and rhegmatogenous retinal detachments [95].

In most of these cases, the use of periocular or intravitreal steroid injections has been for the treatment of acute exacerbations, often in combination with the commencement of systemic treatment to prevent the relapse of disease when the sustained release steroid is exhausted. However, due to their limited duration of effect, this modality of treatment tends not to be used as the sole treatment in chronic disease. However, their use during pregnancy would appear ideal, as they have very little (if any) systemic toxicity and only a limited and finite number of repeated administrations would be needed (if required) during the course of the pregnancy, after which systemic treatments could be reconsidered after delivery. Alternatively, newer forms of sustained release corticosteroid therapy such as Ozurdex (Allergan, Irvine, CA) and Retisert (Bausch and Lomb, Rochester, NY) could also be considered during this time, given their longer durations of effect in chronic active posterior or panuveitis, with similar efficacy to systemic treatment [96, 97].

A suggested approach in the management of patients with chronic uveitis who become pregnant would therefore be the tapering and cessation of systemic treatments during pregnancy, as the activity of the patient's uveitis would be expected to decrease during this time. Any flare-ups of disease could then be managed locally with either topical, sub-Tenons, or intravitreal sustained release corticosteroid as required until delivery. For those with sight-threatening disease, repeated prophylactic local injections could be considered; however, this would be a more contentious approach, given that disease activity is expected to reduce during pregnancy and common side effects such as raised IOP and cataracts are higher in younger patients [81, 94]. Upon delivery, recommencement of systemic agents (being mindful of the patient's breastfeeding status) and closer review of patients would then be recommended, given that uveitis activity is likely to rebound back to prepregnancy levels. For patients with chronic, sight-threatening disease where the cessation of systemic treatment is deemed particularly risky, an alternative option is the use of either the Ozurdex or Retisert sustained release corticosteroid devices. In those patients planning for multiple children, Retisert may be particularly advantageous, given its much longer duration of effect [98].

## 5. Conclusion

The influence of pregnancy on the course of uveitis is a fascinating phenomenon. The general consensus is that uveitis improves during pregnancy, especially from mid pregnancy onwards, while the postpartum period is associated with uveitis activity relapse. This has key implications on the management of pregnant uveitis patients. Clinicians may consider decreasing uveitis medications during pregnancy to minimise medication associated side effects on the foetus. After delivery, followup should also be intensified in anticipation of postpartum relapse. It would be interesting to see if future studies on the mechanisms behind uveitis amelioration in pregnancy would inspire new therapeutic options for uveitis.

## Figures and Tables

**Table 1:** Immunosuppressive drugs in pregnancy and lactation (adapted from reviews on immunomodulatory agents in pregnancy) [59, 62–65].

Class	Side effects on pregnancy and foetus	Recommendations
Corticosteroids		
Prednisolone	(i) Foetal: cleft palate/lip, foetal growth retardation, adrenal suppression, neonate cataract [99, 100] (ii) Maternal: glucose intolerance, hypertension, osteopenia	(i) Food and Drug Administration Category B drug (ii) May be used in pregnancy and breastfeeding (iii) Ideally use prednisolone doses of ≤10 mg/day (iv) May need stress dosing (hydrocortisone/methylprednisolone) at labour, delivery, immediate postpartum period [63, 101] (v) Prednisolone level in milk is <0.1% of the prednisolone dose ingested by the mother Minimise exposure by nursing 4 hours after dose is taken if daily dose exceeds 20 mg [102, 103]

Antimetabolites		
Azathioprine 6-Mercaptopurine	(i) Foetal: the foetal liver lacks the enzyme, inosinate pyrophosphorylase, which converts azathioprine to active metabolites; therefore the fetus is protected from the adverse effects of azathioprine (especially early pregnancy) [104] (ii) Paternal: male fertility and pregnancy do not seem to be affected [105]	(i) Food and Drug Administration Category D drug (ii) Has been used in pregnancy for many years [64] (iii) Ideally use doses <2 mg/kg/day. Consider decreasing dose at 32 weeks [63] (iv) Breastfeeding is not recommended [106]
Methotrexate (MTX)	(i) Foetal: miscarriage, congenital malformations (limb defects, cranial and central nervous system abnormalities) especially in first trimester (ii) Paternal: oligospermia (may be irreversible)	(i) Food and Drug Administration Category X drug (ii) Cease 3 months before conception (male and females), continue folic acid after stopping MTX and during pregnancy (iii) Not considered safe in breastfeeding due to inadequate data
Mycophenolate mofetil (MMF)	(i) Foetal: congenital malformations (distinctive MMF embryopathy), abortions (especially in first trimester) (ii) Paternal: male fertility and pregnancy do not seem to be affected	(i) Food and Drug Administration Category D drug (ii) Avoid in pregnancy (iii) Use of MMF in pregnancy has not been widely studied; however available reports suggest avoiding MMF if possible during pregnancy [107–109] (iv) Cease >6 weeks before conception attempted [63] (v) MMF is often switched to azathioprine during pregnancy [65] (vi) Breastfeeding is not recommended [65]

T-cell inhibitors		
Cyclosporine	(i) Foetal: infant T-, B-, NK-cell development abnormalities [110] (ii) Maternal: renal impairment, hypertension, lymphoma (iii) Paternal: male fertility and pregnancy do not seem to be affected	(i) Food and Drug Administration Category C drug (ii) May be used during pregnancy (iii) Dosage 2.5–5 mg/kg/day—not recommended for use in breastfeeding. However, there have been reports of use in breastfeeding without adverse effects [111]
Tacrolimus	(i) Foetal: risk of congenital malformations and abortions	(i) Food and Drug Administration Category C drug (ii) Insufficient information to recommend use in pregnancy (iii) Avoid breast feeding

Interferon		
Interferon-2a	(i) Foetal: not teratogenic in animal studies	(i) Food and Drug Administration Category C drug (ii) American College of Paediatricians classifies interferon-2a as safe in pregnancy and breastfeeding (iii) However, given the limited data on human studies, it should be avoided in pregnancy ideally

Anti-TNF		
Infliximab Adalimumab Etanercept	(i) Foetal: possible risk of VACTERL (vertebral anomalies, anal atresia, cardiac defects, tracheoesophageal fistula, esophageal atresia, renal anomalies, limb dysplasia). Currently effects are still uncertain [62, 112–115] (ii) TNF antagonists may affect fertility [116]	(i) Food and Drug Administration Category B drug (ii) Not recommended for use in pregnancy and breastfeeding, unless potential benefits outweigh the potential risks [63] (iii) Limited data on infliximab use in lactation, therefore should avoid breastfeeding (iv) Cease infliximab for 6 months before starting breastfeeding

Anti-CD 20 B-cell inhibitor		
Rituximab	(i) Foetal: case reports of granulocytopenia and lymphopenia	(i) Food and Drug Administration Category C drug (ii) Not recommended for use in pregnancy and breastfeeding, unless potential benefits outweigh the potential risks (iii) Cease 1 year before attempting conception

Interleukin-1 receptor antagonist		
Anakinra	(i) Foetal: no toxicity demonstrated in animal studies	(i) Food and Drug Administration Category B drug (ii) Only use in pregnancy and lactation if needed to suppress disease activity

Alkylating agents		
Cyclophosphamide	(i) Foetal: congenital malformation (craniofacial and distal limb defects), developmental delay [117] (ii) Maternal: infertility, amenorrhoea, ovarian failure (iii) Paternal: oligospermia (may be irreversible) [118–120]	(i) Food and Drug Administration Category X drug (ii) Absolutely contraindicated in the first trimester but may be used in latter half of pregnancy [64] (iii) Cease 3 months before attempting conception (iv) Contraindicated in breastfeeding [121]

Dihydrofolate reductase inhibitor		
Sulfasalazine	(i) Foetal: kernicterus, agranulocytosis, no significant increase in congenital abnormalities [62, 122–124] (ii) Paternal: oligospermia (reversible)	(i) Food and Drug Administration Category B drug (ii) Probably safe for use in pregnancy [124] and breastfeeding [125, 126]

Intravenous Immunoglobulin therapy		
		(i) Food and Drug Administration Category C drug (ii) Good safety profile in use during pregnancy (in studies on autoimmune conditions, other than uveitis)
